# ABCC transporters mediate insect resistance to multiple Bt toxins revealed by bulk segregant analysis

**DOI:** 10.1186/1741-7007-12-46

**Published:** 2014-06-09

**Authors:** Youngjin Park, Rosa M González-Martínez, Gloria Navarro-Cerrillo, Maissa Chakroun, Yonggyun Kim, Pello Ziarsolo, Jose Blanca, Joaquin Cañizares, Juan Ferré, Salvador Herrero

**Affiliations:** 1 Department of Bioresource Sciences, Andong National University, Andong 760-749, Korea; 2 Department of Genetics, Universitat de València, Dr Moliner 50, 46100 Burjassot, Spain; 3 Institute for Conservation & Improvement of Valentian Agrodiversity (COMAV), Polytechnic University of Valencia, Valencia, Spain

**Keywords:** Bt resistance, ABCC2 transporter, Cry toxins

## Abstract

**Background:**

Relatively recent evidence indicates that ABCC2 transporters play a main role in the mode of action of *Bacillus thuringiensis (*Bt) Cry1A-type proteins. Mapping of major Cry1A resistance genes has linked resistance to the *ABCC2* locus in *Heliothis virescens*, *Plutella xylostella*, *Trichoplusia ni* and *Bombyx mori*, and mutations in this gene have been found in three of these Bt-resistant strains.

**Results:**

We have used a colony of *Spodoptera exigua* (Xen-R) highly resistant to a Bt commercial bioinsecticide to identify regions in the *S. exigua* genome containing loci for major resistance genes by using bulk segregant analysis (BSA)*.* Results reveal a region containing three genes from the *ABCC* family (*ABBC1*, *ABBC2* and *ABBC3*) and a mutation in one of them (*ABBC2*) as responsible for the resistance of *S. exigua* to the *Bt* commercial product and to its key Spodoptera-active ingredients, Cry1Ca. In contrast to all previously described mutations in ABCC2 genes that directly or indirectly affect the extracellular domains of the membrane protein, the ABCC2 mutation found in *S. exigua* affects an intracellular domain involved in ATP binding. Functional analyses of *ABBC2* and *ABBC3* support the role of both proteins in the mode of action of *Bt* toxins in *S. exigua.* Partial silencing of these genes with dsRNA decreased the susceptibility of wild type larvae to both Cry1Ac and Cry1Ca. In addition, reduction of *ABBC2* and *ABBC3* expression negatively affected some fitness components and induced up-regulation of *arylphorin* and *repat5*, genes that respond to *Bt* intoxication and that are found constitutively up-regulated in the Xen-R strain.

**Conclusions:**

The current results show the involvement of different members of the *ABCC* family in the mode of action of *B. thuringiensis* proteins and expand the role of the ABCC2 transporter in *B. thuringiensis* resistance beyond the Cry1A family of proteins to include Cry1Ca.

## Background

Genes coding for insecticidal proteins from the bacterium *Bacillus thuringiensis (Bt)* have been transferred to plants to protect them from the attack of insect pests [1,2] and the global impact of this strategy has been unprecedented in plant biotechnology. Unfortunately, and similarly with the history of chemical insecticides, insects have developed resistance to *B. thuringiensis* crystal proteins (Cry proteins) when exposed to either foliar *B. thuringiensis* sprays or transgenic crops (Bt-crops) [3-5]. The long lasting benefits of *B. thuringiensis* insecticides and Bt-crops will mostly depend on the understanding of the mode of action of Cry proteins and of the biochemical and genetic bases of insect resistance.

All *B. thuringiensis* crystal proteins (Cry proteins) which are toxic to target insects share general traits in their mode of action: solubilization in the midgut, activation by gut proteases, binding to membrane surface proteins (generally referred to as ‘receptors’) [6,7]. Susceptible insects have epitopes in their membrane receptors that are recognized by sequences of the Cry proteins. This is probably the main reason why Cry proteins are not harmful to non-target insects [8]. The key role of membrane receptors has also become evident when studying the biochemical basis of insect resistance to Cry toxins. Most cases of high level resistance to Cry1A proteins have been shown to be due to a decrease of toxin binding to midgut receptors [9].

Two approaches have been undertaken to characterize membrane proteins involved in the toxicity of Cry proteins. Formerly, the approach was biochemical, and it allowed for the characterization of receptors such as cadherin, aminopeptidase N (APN), and alkaline phosphatase (ALP) [10], among others. The occurrence of such a variety of receptors has been explained by integrating them in a ‘toxicity pathway’ that implies their participation in a sequential way [7]. In the most accepted model, binding to cadherin is required for the Cry proteins to oligomerize and then bind to other receptors, such as APN and ALP, which favor toxin insertion into the membrane. There is evidence supporting the participation of cadherin and APN in the toxicity of Cry proteins [11,12]. Furthermore, mutations conferring resistance to proteins from the Cry1A family have been found in the *cadherin* gene from *Heliothis virescens*[13], *Helicoverpa armigera*[14,15] and in *Pectinophora gossypiella*[16]. Similarly, mutations or reduced expression of *apn* genes have been found in resistant insects from *Spodoptera exigua*[17], *Diatraea saccharallis*[18] and *Trichoplusia ni*[19].

Another membrane protein, identified as an ABCC2 transporter, was found related to the mode of action of Cry1A-type proteins using a genetic approach to identify major genes involved in resistance in *H. virescens*[20]. Later on, mutations in the *ABCC2* gene were found in Cry1A resistant colonies from three other insect species: *Plutella xylostella*[21], *T. ni*[21], and *Bombyx mori*[22]. Further evidence for the involvement of the ABCC2 transporter in Cry1A toxicity came from functional studies with transfected cultured cells [23]. Although mutations in either the *cadherin* or in the *ABCC2* gene alone may suffice to render high levels of resistance, it seems clear that the occurrence of both functional genes is necessary for the full toxicity of Cry1A proteins [20,23]. Although the binding of Cry1A proteins to the ABCC2 transporter has never been directly shown, it is thought that the ABCC2 transporter transiently binds the Cry oligomer and mediates its insertion from the APN or ALP receptors to the membrane [24].

In a previous work we have reported an *S. exigua* strain (Xen-R) with high levels (>1,000-fold) of resistance to a *B. thuringiensis*-based pesticide, Xentari™ [25]. Expression of about 600 midgut genes was analyzed by DNA-macroarray in order to find differences in midgut gene expression between susceptible and resistant insects. Among the differentially expressed genes, *repat* and *arylphorin* were identified and their increased expression was correlated with *B. thuringiensis* resistance. We also found overlap among genes that were constitutively over-expressed in resistant insects with genes that were up-regulated in susceptible insects after exposure to Xentari™, suggesting a permanent activation of the response to Xentari™ in resistant insects. However, we could not determine the exact resistant gene or genes and the molecular mechanism responsible of the high levels of resistance to *B. thuringiensis* in these insects.

In the present paper we used bulk segregant analysis (BSA) based on high-throughput sequencing methodologies (also known as ‘next generation sequencing’ or NGS) [26,27] to identify regions in the *S. exigua* genome containing loci for major resistance genes in Xen-R strain [25]*.* The results reveal two *ABCC* genes (*ABBC2* and *ABBC3*) involved in the mode of action of *B. thuringiensis* toxins and a mutation in one of them (*ABBC2*) as responsible for the resistance of *S. exigua* to a *B. thuringiensis* commercial product and to its main active ingredients, Cry1Ca and Cry1A toxins. The current results expand the role of the ABCC2 transporter in *B. thuringiensis* resistance beyond the Cry1A family of proteins to include Cry1Ca.

## Results

### Construction of backcross families for resistance mapping

Mapping of resistance was approached by mixing the resistant and susceptible genomes followed by selection for resistance after one round of backcrosses. At the concentration of the Xentari™ product used, Xen-R showed incomplete resistance because 65% of the larvae did not survive the treatment. The same treatment killed 100% of FRA and F1 larvae, indicating that resistance was completely recessive at the diagnostic concentration. After crossing F1 and Xen-R adults and selection with the discriminant concentration of the Xentari™ product, three families resulting from independent backcrosses were randomly selected for the BSA. Mortality in the treated samples was 87% for BC1, 89% for BC3 and 84% for BC4. Considering that the baseline mortality for Xen-R was 65%, the mortality in the three backcross (BC) families is in good agreement with resistance being due to one major locus. Survivors from the three BC families were pooled (Sel-BC sample) and used for the BSA.

### New generation sequencing, assembly and mapping

Parental samples were differentially tagged and sequenced in a single Hi-Seq2000 paired-end (PE) line and Sel-BC in a second line. A summary of the sequencing and assembling process is reported in Table 1. These reads (GenBank SRA accession: SRS514057, SRS514097, and SRS514098), along with the 445,524 sequences from the 454-sequencing and the previously published 6,212 Sanger sequences [28], were assembled using Trinity, yielding a total of 138,989 unigenes and 85,811 gene clusters with an average length of 881 bp, with a total length 119 Mb. These unigenes were filtered to remove small, non-highly expressed anonymous unigenes. Finally, 96,676 unigenes and 54,478 gene clusters were kept, with an average size of 1,124.25 and a total length of 109 Mb (GenBank BioProject 219058). These unigenes were compared using bi-directional Blast search with *Bombyx* and *Drosophila* proteins. For 10,421 and 15,116 unigenes, orthologs were identified in *Drosophila* and silkworm, respectively (Table 1, and Additional file 1). All reads were mapped against a filtered reference transcriptome built with the longest unigene of each gene cluster. The proportion of the total sequences which could be properly mapped was 87.27%, 88.19% and 88.57% from Sel-BC, FRA and Xen-R, respectively. The average coverage was 763.9x.

**Table 1:** NGS results for the different samples and transcriptome statistics

**Sample**	**Raw reads**	**Cleaned reads**	**% mapped sequences**
Xen-R	179,215,722	168,518,208	88.57
FRA	200,970,866	187,779,324	88.19
BC-sel	388,370,500	360,354,916	87.27
**Transcriptome**	**Non-filtered**	**Filtered**	
Unigenes	138,989	96,676	
Gene clusters	85,811	54,478	
N50	1,781	2,016	
Size (Mb)	119	109	
*Drosophila* orthologs	-	10,421	
Silkworm orthologs	-	15,116	

NGS, next generation sequencing.

### Bulk segregant analysis

**Figure 1:**
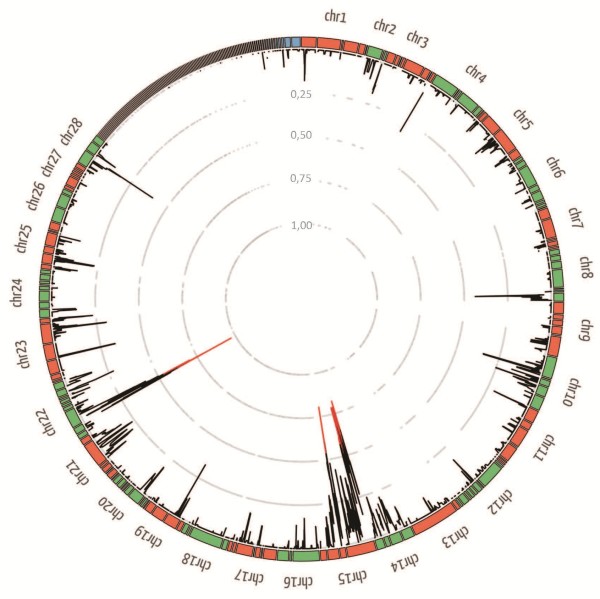
**Schematic representation of the BSA index for the SNPs in the *S. exigua *unigenes with orthologs into the silkworm genome.** Each data point (from 0 to 1) is the average index for three consecutive orthologs calculated using overlapping rolling-windows (BSA index for each unigene individually is provided in Additional file 3). Average index >0.60 are painted in red. BSA, bulk segregant analysis; SNPs, single nucleotide polymorphism.

Using Varscan, 437,815 single nucleotide polymorphisms (SNPs) and 80,246 indels were identified [see Additional file 2]. To select unigenes with a biased allele distribution in the Sel-BC sample, the following index was calculated: ratio of SNPs with the Xen-R allele fixed in Sel-BC (frequency higher than 0.95) against the total number of SNPs. This index ranged from 0 to 1. Unigenes with an index close to 1 had a bias towards the allele present in the Xen-R parental. This index was plotted along with the *Bombyx* genome (Figure 1) by using the position of the respective *Bombyx* orthologs. A total of 978 unigenes [see Additional file 3]. With a silkworm ortholog were available. Two wide *Bombyx* genomic regions with a high bias towards the resistant allele were detected: a wider region in chromosome 15 and another smaller region in chromosome 22. The region in chromosome 15 spanned from 64,363 to 3,068,277 of nscaf2888 scaffold and contained homologs to 64 *S. exigua* unigenes. The region in chromosome 22 spread from 67,364 to 1,716,412 of nscaf1681 scaffold and contained homologs to 42 *S. exigua* unigenes.

Detailed analysis of the chromosome 22 region did not reveal genes potentially involved in the mode of action of *B. thuringiensis* or other entomopathogens. Moreover, we could not identify any SNPs associated with Sel-BC or Xen-R insects having a major effect (for example, nonsense mutation) on the codified proteins when compared to the alleles in FRA insects.

**Figure 2:**
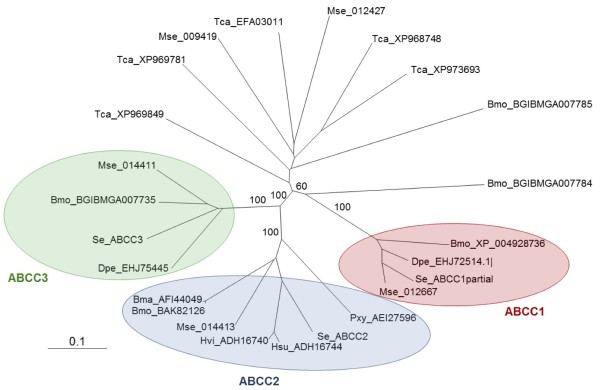
**Phylogenetic analysis and ortholog asignment of *S. exigua *ABCC transporters.** Neighbor-joining consensus tree of representative members of ABC transporters from Lepidoptera and *Tribolium castaneum* (Coleoptera). Nomenclature for each member is based on the name of the species (Bmo: *Bombyx mor*i; Se: *Spodoptera exigua*; Dpe: *Danaus plexippus*; Bma: *Bombyx mandarina*; Hvi: *Heliothis virescens*; Hsu: *Heliothis subflexa*; Mse: manduca sexta; Tca: *Tribolium castaneum*) followed by their accession number from the National Center for Biotechnology Information (NCBI), Silkdb and Manduca Base (Agripest) databases. Sequences were selected based on their homology to the ABCC1-3 transporters from the linkage region in *B. mori*. Some homologs in *T. cataneum* were selected as a kind of outgroup. For the sake of clarity bootstrap values are only reported for the branches identifying the main ABCC subgroups. NCBI, National Center for Biotechnology Information.

**Figure 3:**
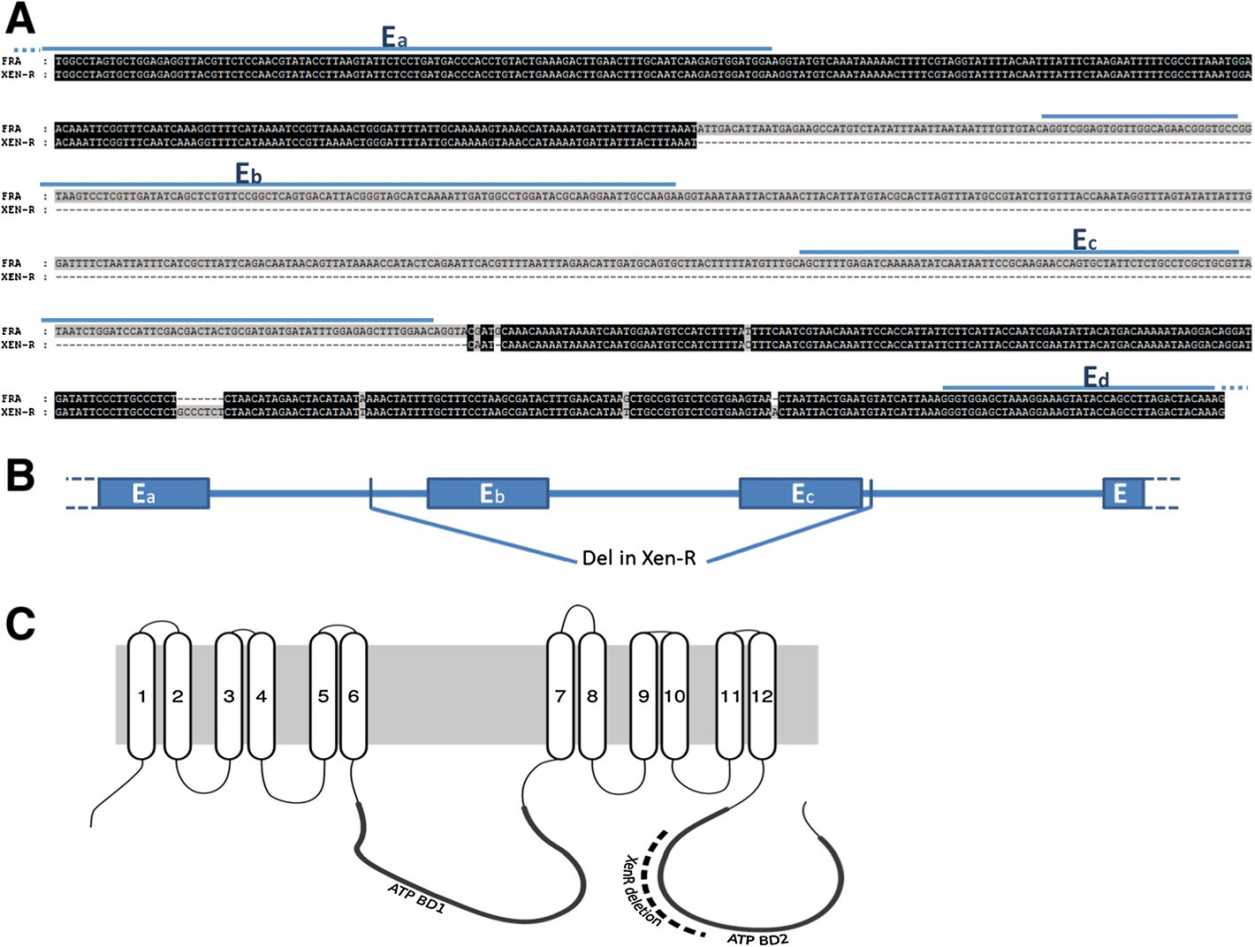
**Localization of the deletion in resistant insects into the *ABCC2 *gene genomic region (A) schematically represented at the genomic region (B) and on the hypothetical protein (C).** Sequences were obtained by PCR amplification of the reported region using genomic DNA from the Xen-R and FRA strains. ‘E’ labels are indicative of the predicted exons in the reported region. The dashed line indicates the deletion in the Xen-R insects. PCR, polymerase chain reaction.

Chromosomal regions from several insect species homologous to a region in chromosome 15 in *B. mori* have been previously associated with resistance to *B. thuringiensis* Cry1A toxins [20-22]. Such a *B. mori* region contains several members of the *ABCC* family. Detailed analysis of unigenes from *S. exigua* in that region identified three *ABCC* genes, which we named *ABCC1*, *ABCC2* and *ABCC3* according to a phylogenetic analysis with representative members of ABC transporter genes from other Lepidoptera and Coleoptera (Figure 2). The *S. exigua ABCC2* and *ABBC3* genes codify for a 12-transmembrane domain protein [see Additional file 4: Figure S1]. Although our transcriptome did not contain the complete *ABCC1* unigene sequence, comparison with other *ABCC1* genes suggests that it codifies for a protein with a topology similar to that of other ABCC1 transporters. Allelic comparison of unigenes in that region revealed a major mutation in the *ABCC2* gene: a deletion of 246 nucleotides (82 amino acids) in the unigene from the Xen-R and Sel-BC samples, but not from the FRA sample. Despite the high coverage for that gene in the Sel-BC sample, no single read was observed mapping into the deleted region. The deleted region included a fragment of the predicted ATP binding domain 2 in the hypothetical protein (Figure 3C). Further analysis of the genomic region revealed a deletion of about 500 nucleotides extending over two exons (Figure 3).

Based on these results, we decided to focus on the possible function of the ABCC2 and ABCC3 proteins, especially on their role in the susceptibility to *B. thuringiensis* toxins and the effect of the ABCC2 mutation on the binding of Cry1Ca toxin to the insect midgut.

#### Expression profiles of SeABCC2 and SeABCC3 genes in S. exigua

**Figure 4:**
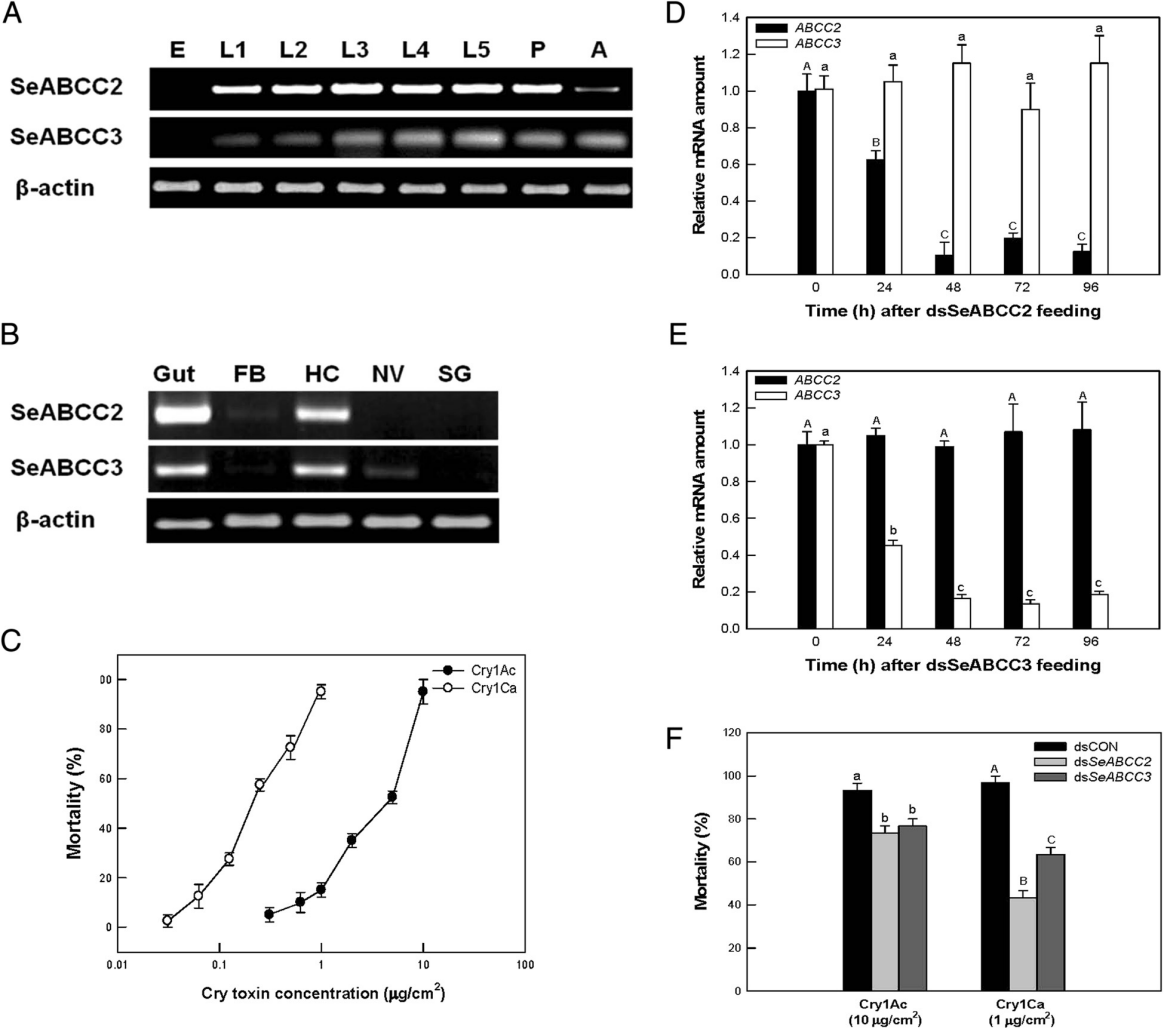
**Expression patterns of *SeABCC2 *or *SeABCC3 *gene in different developmental stages (A) and in different tissues (B) of the fifth instar larvae in *S. exigua*.** ‘E’, ‘L1 – L5’, ‘P’, and ‘A’ represent egg, larval instars, pupa, and adult, respectively. ‘FB’, ‘HC’, ‘NV’, and ‘SG’ represent fat body, hemocytes, nerve, and salivary gland, respectively. Expression of β-actin confirms the integrity of cDNA preparation. Toxicity comparison of Cry1Ac and Cry1Ca protoxins against third instar larvae of *S. exigua***(C)**. Each bioassay was conducted with ten larvae per replicate and three replicates per concentration. Mortality was scored at five days post-treatment. Suppression of *SeABCC2* or *SeABCC3* gene expression using a specific double-stranded RNA (ds*SeABCC2* or ds*SeABCC3*) in the whole body of *S. exigua* larvae at different periods (**D** and **E**, respectively). *SeABCC2 or SeABCC3* expression was analyzed by qRT-PCR. Each treatment was independently replicated three times. Different letters above standard deviation bars indicate significant differences among means at Type I error = 0.05 (LSD test). The effect of suppression of *SeABCC2* or *SeABCC3* gene expression on susceptibility of the third instar *S. exigua* to Cry1Ac or Cry1Ca protoxins **(F)**. A viral gene (ORF302)-specific dsRNA (dsCON) was used for a dsRNA control. At 24 hours after dsRNA treatment, the larvae were exposed to Cry1Ac (10 μg/cm^2^) or Cry1Ca (1 μg/cm^2^) protoxins treated by the leaf-dipping method. Mortality was assessed at five days after Cry toxin treatment. Each treatment was replicated three times. Each replication used 10 larvae. Different letters above standard deviation bars indicate significant difference among means at Type I error = 0.05 (LSD test). dsRNA, double-stranded RNA; LSD, least significant difference; qRT-PCR, quantitative reverse transcriptase-polymerase chain reaction.

Gene expression of *SeABCC2* and *SeABCC3* was analyzed by reverse transcriptase-polymerase chain reaction (RT-PCR) in different developmental stages of *S. exigua* and in different tissues of fifth instar larvae. *SeABCC2* and *SeABCC3* were expressed in all larval instars and in the adult stage although at early stages *SeABCC2* was apparently more strongly expressed than *SeABBC3* (Figure 4A). Regarding tissue distribution (Figure 4B), *SeABCC2* was highly expressed in the gut and hemocytes; in addition, the sample from the fat body gave a very faint amplification band. *SeABCC3* was also highly expressed in the gut and hemocytes and faint amplification bands were obtained in samples from the fat body and nerve, but not from the salivary glands.

#### Effect of SeABCC2 and SeABCC3 suppression on the susceptibility of S. exigua to Cry1Ac and Cry1Ca

To study the role of these proteins in the susceptibility of *S. exigua* to *B. thuringiensis* toxins, *SeABCC* genes were silenced by RNA interference (RNAi) and the suppressed larvae were tested for their susceptibility to two different Cry1 toxins. Prior to performing bioassays with double-stranded RNA (dsRNA)-treated larvae, the susceptibility of non-treated *S. exigua* third instar larvae to Cry1Ac and Cry1Ca protoxins was determined under our experimental conditions. As expected, Cry1Ca was significantly more toxic than Cry1Ac, with LC_50_ values of 0.24 and 4.8 μg/ml, respectively. The toxin concentrations that yielded mortality between 90% and 100% were 10 and 1 μg/cm^2^ for Cry1Ac and Cry1Ca, respectively (Figure 4C). These toxin concentrations were chosen for subsequent experiments with dsRNA.

Expression of *SeABCC2* and *SeABCC3* started to decrease after 12 hours of dsDNA ingestion, but the suppression was more drastic after 48 hours and lasted at least up to 72 hours in both cases (Figure 4D-E). Cross-suppression between the two genes was discarded by estimation of *SeABCC2* expression in ds*SeABCC3*-treated insects and *vice versa* (Figure 4D-E)*.* When *S. exigua* larvae were treated with either ds*SeABCC2* or ds*SeABCC3* and then exposed to 10 μg/cm^2^ of Cry1Ac or 1 μg/cm^2^ of Cry1Ca, they showed a significant decrease in susceptibility to these two toxins (Figure 4F). In particular, silencing of *SeABCC2* highly decreased Cry1Ca toxicity to *S. exigua* compared with silencing of *SeABCC3*. Under these conditions, control larvae suffered about 92% and 95% larval mortality to Cry1Ac or Cry1Ca, respectively.

### Binding of Cry1Ca to brush border membrane vesicles from resistant and susceptible larvae

**Figure 5:**
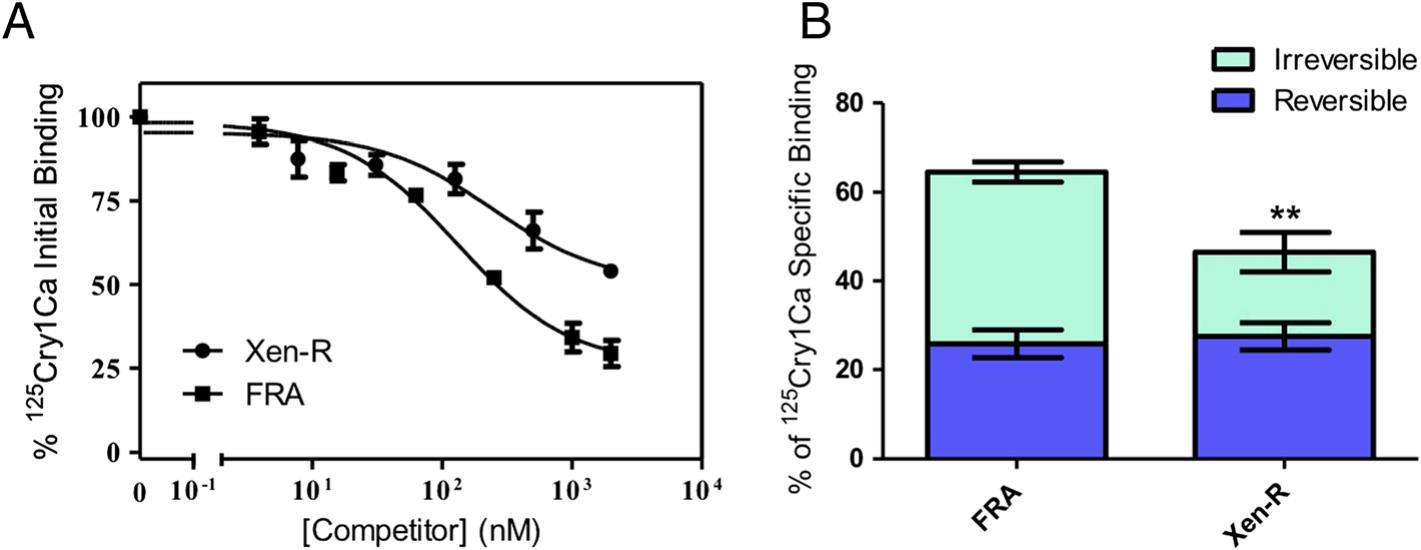
**Binding of **^**125**^**I-labeled Cry1Ca to *S. exigua *BBMV. (A)** Homologous competition. ^125^I-labeled Cry1C was incubated with BBMV from susceptible (FRA) or resistant (Xen-R) insects in the presence of increasing concentrations of unlabeled Cry1C toxin. **(B)** Dissection of total binding into their reversible and irreversible components. Contribution of both components to the binding of ^125^I-labeled Cry1Ca to BBMV from susceptible and resistant insects were compared. T-test (*P* = 0.7244 for the reversible component) (*P* = 0.0170 for the irreversible component). BBMV, brush border membrane vesicles.

Since mutations in the ABCC2 transporters have been proposed to confer resistance to insects from different species that lacked binding of one or more Cry1A proteins [20,21] binding of ^125^I-labeled Cry1Ca was tested in susceptible and resistant insects to see whether binding was altered in the latter (Figure 5). Competition binding curves indicated that resistant insects had a high contribution of non-specific binding (the binding that cannot be displaced with high concentrations of competitor) to the total binding (Figure 5A). The equilibrium binding parameters did not differ significantly between the two types of insects and the only significant differences were found in the concentration of binding sites (Table 2). Specific binding of Cry toxins to their membrane receptors consists of a reversible binding component and an irreversible binding component associated with the toxin insertion into the membrane [29]. To search for additional possible differences in binding, an experiment was designed to dissect the total specific binding into its reversible and irreversible components. The results showed a significant decrease in the irreversible component of the specific binding in resistant insects as compared with susceptible insects (Figure 5B). However, the reversible component was similar in both cases.

**Table 2:** **Equilibrium dissociation constant (*K***_***d***_**) and concentration of binding sites (*R***_***t***_**) of Cry1Ca binding to BBMV from Xentari™ susceptible (FRA) and resistant (Xen-R) *S. exigua *** **larvae**

**Colony**	**Mean ± SEM**^***a***^	
	***K***_***d ***_**(nM)**	***R***_***t ***_**(pmol/mg)**
FRA	14 ± 2	5.9 ± 0.6
Xen-R	28 ± 13	2.85 ± 1.13

^*a*^From two independent replicates. BBMV, brush border membrane vesicles; SEM, standard error of the mean.

#### Fitness-cost associated with the silencing of SeABCC2 or SeABCC3 expression

**Figure 6:**
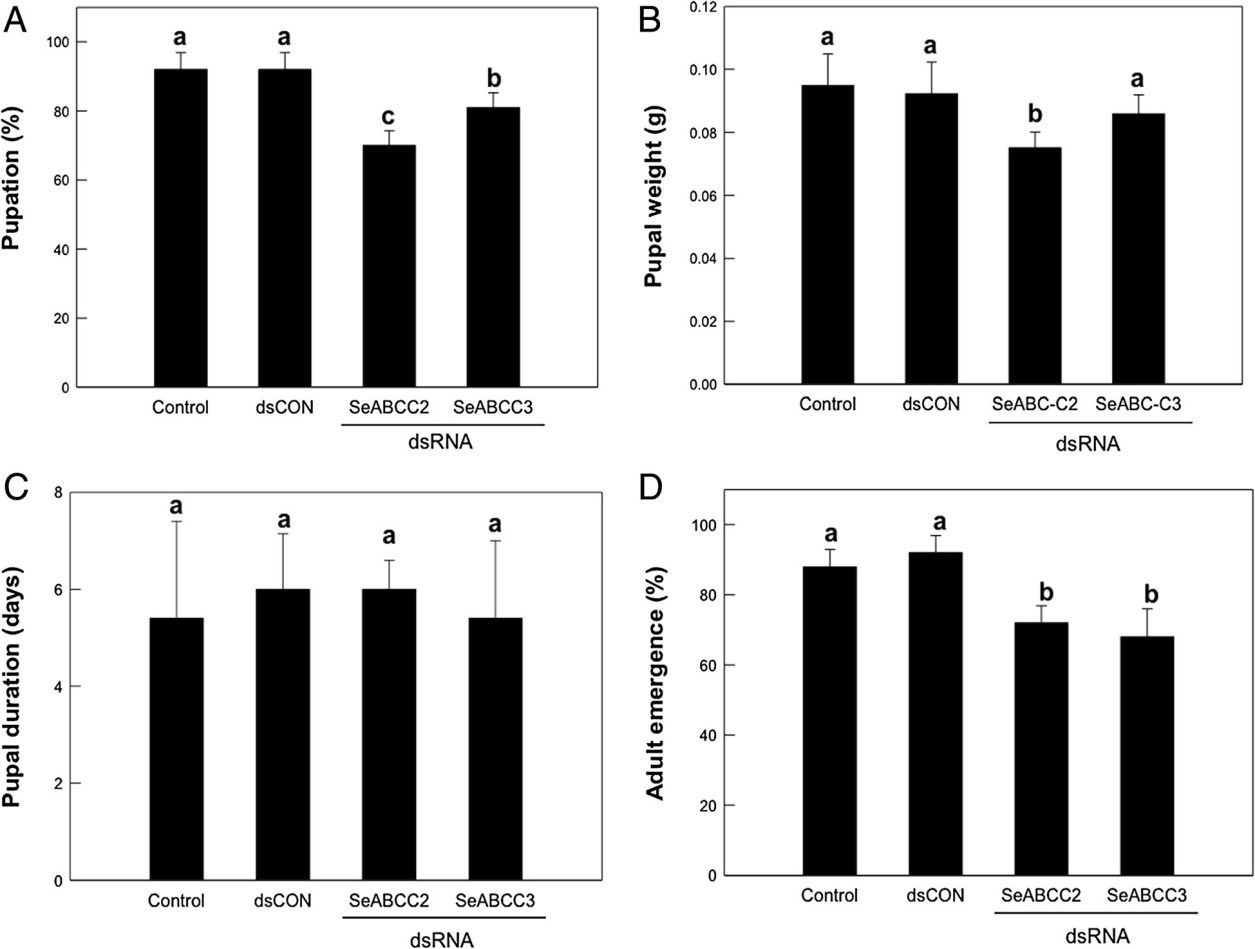
**Effect of suppression of *SeABCC2 *or *SeABCC3 *gene expression on pupation (A, B, and C) and adult emergence (D) of *S. exigua*.** The expression of *SeABCC2* or SeABCC3 gene was suppressed by feeding dsSeABCC2 or dsSeABCC3 to newly molted second instar larvae. A viral gene (ORF302)-specific dsRNA (dsCON) was used for a dsRNA control. Each treatment was replicated three times. **(A)** Pupation, **(B)** pupal weight, **(C)** pupal duration and **(D)** adult emergence after dsRNA feeding were measured. Each replicate used 10 larvae. Different letters above standard deviation bars indicate significant differences among means at Type I error = 0.05 (LSD test). dsRNA, double-stranded RNA, LSD, least significant difference.

The effect of silencing of either *SeABCC2* or *SeABCC3* on the fitness of the insects was also measured. Reduction in the expression level of both genes had a statistically significant influence on both pupation and adult emergence. Feeding dsRNA specific to either *SeABCC2* or *SeABCC3* reduced the percentage of pupation to 70% and 81%, respectively (Figure 6A) and the percentage of adult emergence to 72% and 68%, respectively (Figure 6D). Treatment of ds*SeABCC2* also had a significant effect on the pupal weight, although that of ds*SeABCC3* did not (measured at the second day of pupal stage) (Figure 6B). In contrast, none of the treatments had a significant effect on the duration of the pupal stage, which in all cases took five to six days from the first day of pupation till adult emergence (Figure 6C).

#### Overexpression of arylphorin and repat5 genes induced by SeABCC2 and SeABCC3 suppression

Since both *arylphorin* and *repat5* have been reported to be overexpressed in the Xen-R insects [25], it was of interest to determine whether the silencing of *SeABCC2* or *SeABCC3* could have an effect on the expression of these genes. The results show, in agreement with previous reports, that when either the *SeABCC2* or *SeABCC3* gene is silenced, both *arylphorin* and *repat5* genes increased their expression as compared with that of the controls [see Additional file 5: Figure S2].

## Discussion

Forward genetics has been useful in shedding light on new resistance mechanisms to Cry1A proteins in insects. Relatively recently, the role of the ABCC2 transporter has been made evident in the mode of action of Cry1A proteins through linking resistance to mutations in this membrane protein in four different insect species [24]. We have successfully adapted standard BSA procedure to our particular conditions: 1) since we could not easily discriminate susceptible insects in the BC offsprings we have only used one of the bulk extremes (the resistant insects). As a consequence, evidence of linkage to resistance was not deduced by the comparison of the frequency of the markers in the two bulks, but by comparing their frequency deviation from the expected 50% and their deduced genomic localization. 2) Due to the absence of genomic information on *S. exigua,* the resistance gene has been positioned and deduced using the genome of a related Lepidoptera, *B. mori.* 3) Genetic markers have been discovered by RNA sequencing instead of direct examination of genomic DNA. BSA based on RNA information could introduce a certain bias because the cDNA contribution to the bulk can be allele specific. To prevent or minimize such a possibility the signal has been detected at the genomic level and not at the gene level, thus averaging out the possible bias present in one particular gene. Our approach using NGS and BSA applied to *S. exigua* has pinpointed two genomic regions with high segregation distortion that have a high probability of containing genes involved in the resistance to *B. thuringiensis*. BSA has been previously employed for the identification of a pesticide resistance gene [30]; however, as far as we know, this is the first report of successful mapping of insect resistance genes by combining asymmetric BSA with RNA sequencing.

One of these two regions contained three genes coding for ABC type C transporters, including the *ABCC2* gene along with *ABCC1* and *ABCC3* genes. The sequences of the *ABCC2* and *ABCC3* genes coded for 12-transmembrane domain proteins. Most interestingly, the *ABCC2* allele from the resistant insects contained a deletion that eliminates a cytoplasmic portion of the protein affecting one of the two ATP binding regions, making this gene a candidate for the resistance in the Xen-R colony. The second region, located in chromosome 22, did not reveal genes potentially involved in the mode of action of *B. thuringiensis*, although we cannot discard the presence of additional genes in such a region contributing to the resistance in the Xen-R insects. Alternatively, synteny is not complete in Lepidoptera and it could be possible that both regions are located in the same chromosome in *S. exigua*.

To determine the role of the ABCC2 transporter, and that of its closest paralog ABCC3, in the mode of action of Cry1 proteins, their developmental expression and tissue distribution has been studied. They both were expressed in the larval midgut, in agreement with the expected characteristics from membrane proteins that must interact with Cry1 proteins toxic to larvae. However, at early stages of development, when insects are more susceptible to *B. thuringiensis,* the expression levels of ABCC3 are lower than those of ABCC2, suggesting that the latter may play a more relevant role in the mode of action of *B. thuringiensis* toxins. A more direct approach to the involvement of ABCC2 and ABCC3 in the toxicity of Cry proteins was to measure the susceptibility to Cry1Ac and Cry1Ca in larvae where the expression of these two transporters had been decreased by feeding dsRNA. With both toxins, mortality significantly decreased when the expression of either ABCC2 or ABCC3 was reduced. This result shows that both transporters are involved in the toxicity of these two Cry1 proteins and that, in theory, mutations in either one could result in resistance to these two toxins. Since the sequence homology between ABCC2 and ABCC3 is higher than 70%, it is likely that both proteins could serve as receptors for Cry1 toxins. ABCC1, although not as similar to ABCC2 as ABCC3, cannot be discarded at this point as also playing a role in the mode of action of *B. thuringiensis*. However, the most direct evidence of the involvement of ABCC transporters in the mode of action of *B. thuringiensis* toxins in *S. exigua* is that resistant insects, but not susceptible controls, carried a mutated allele of the *ABCC2* gene.

All previous reports relating the ABCC2 transporter to resistance to Cry1A proteins have found mutations in the *ABCC2* gene that affected the extracellular domains in different ways. In *H. virescens*, the mutation consisted of a 22-bp deletion in exon 2, which would result in a truncated product of 99 amino acids instead of the 1,339 amino acids in the wild type protein [20]. In *P. xylostella*, the *ABCC2* gene had a 30-bp deletion in exon 20, which was predicted to remove the final transmembrane domain with the result of positioning the C-terminal end of the protein in the extracellular space instead of its natural location inside the cell [21]. In *B. mori*, the mutation added an extra tyrosine residue in the second extracellular loop of the ABCC2 transporter [22]. So far, the mutation in the *ABCC2* gene conferring resistance in *T. ni* has not yet been elucidated [21]. Binding studies with the above resistant colonies showed that insects with the ABCC2 mutation lacked binding of Cry1Ab and Cry1Ac in the case of *H. virescens* and *P. xylostella*[20,31]. The same lack of binding of these two toxins was found in the *T. ni* resistant colony [32]. However, no apparent alteration of Cry1Ab binding (Cry1Ac was not tested) was found in the *B. mori* resistant insects as compared to susceptible controls [22]. As has been hypothesized elsewhere [24], it is very likely that the extra tyrosine residue in the extracellular loop does not prevent binding of Cry1Ab, although it could confer resistance by altering the transporter function and decreasing the probability of the toxin being drawn into the membrane.

The mutation we have found in the *S. exigua* resistant insects is the first reported mutation that does not affect, directly or indirectly, the extracellular ABCC2 domains, but affects the intracellular C-terminal part of the protein, which contains the second ATP binding domain. If the above model of the involvement of the ABCC2 transporter in the mode of action of Cry1 toxins is correct, we should not expect any decrease in toxin binding since the extracellular domains of the protein are not altered, and the intracellular deletion does not seem to appreciably affect the conformation of the rest of the protein. Similarly to *B. mori*, resistance would be conferred by altering the transporter function affecting its role in inserting the toxin into the membrane [24]. Our binding data completely agree with this hypothesis. First, no significant difference was found regarding the affinity of the toxin to bind BBMV, and only a small difference was found between resistant and susceptible insects regarding the concentration of binding sites. Second, alteration of the transporter ability to draw the toxin into the membrane would specifically affect the irreversible component of binding, and this is precisely what our results indicate. The involvement of the ABCC2 transporter in the resistance to *B. thuringiensis* in *S. exigua* adds a new species in which the role of this gene in resistance to Cry proteins has been demonstrated.

Silencing of ABCC2 and ABCC3, in addition to allowing us to confirm the role of these receptors in the mode of action of *B. thuringiensis*, has enabled the study of the fitness cost associated with the absence of these proteins. Although dsRNA feeding could not achieve complete knock-out of gene expression to emulate a resistance gene, partial silencing revealed clear effects on insect fitness. Fitness cost has been associated with resistance in most of the *B. thuringiensis* resistant strains [33]. According to our results, field selection of ABC-related resistance alleles can be counteracted by the reduced fitness of resistant insects versus susceptible insects in areas not exposed to *B. thuringiensis* products (that is, refuges) and, in this way, contribute to delay the development of resistance.

Previous studies on the Xen-R strain revealed that the insects, in the absence of exposure to *B. thuringiensis*, had a high level of expression of certain genes (*arylphorin* and *repat5* among them) that are up-regulated in susceptible insects after *B. thuringiensis* intoxication [25]. For this reason we found it interesting to determine whether a decrease in the expression, or a malfunction, of the SeABCC2 or SeABBC3 transporters could trigger the up-regulation of such genes. Our results reveal that silencing of *SeABCC2* or *SeABCC3*, in susceptible insects, induces the expression of *arylphorin* and *repat5*. Such a result suggests that higher expression levels of these genes in the Xen-R insects are induced by the mutation in the SeABCC2 receptor. Additionally, up-regulation of these genes in response to *B. thuringiensis* intoxication might be induced by the interaction of the toxins with the ABCC receptors, perhaps because binding of the toxin would probably block their physiological function. Other studies have shown that *arylphorin* and *repat5*, as well as other members of the *repat* superfamily, are induced in response to intoxication with several types of toxins in *Spodoptera spp.*[34-36]. It would be of interest to check whether other resistant strains with mutations in the ABCC2 transporter overexpress these genes and to explore the possibility of using such a phenotype as a marker to monitor for the presence of ABC-related mutations in the field.

Also worth noting is the fact that the *ABCC2* mutation in *S. exigua* apparently confers resistance not only to Cry1A proteins but to Cry1Ca as well. Although Xentari™ contains Cry1A and Cry1Ca as major Cry components [37], *S. exigua* larvae are susceptible to Cry1Ca but relatively tolerant to Cry1A proteins [38]. Therefore, resistance to Xentari™ in Xen-R implies resistance to the Cry1Ca key component. Another indirect piece of evidence that mutations in the ABCC2 transporter may be involved in resistance to Cry1 toxins other than those of the Cry1A family was recently reported in *P. xylostella*[39]. Cry1A resistant insects with a mutation in the *ABCC2* gene were cross-resistant to Cry1Fa and they lacked binding of Cry1A proteins and Cry1Fa to BBMV [39]. In this insect species, it was already known that Cry1Fa shares binding sites with Cry1A proteins [40].

Fifteen *ABCC* genes have been annotated on the *B. mori* genome [41,42]. It would be interesting to test whether additional members of such a family in Lepidoptera, but also in other insect orders, could be involved in modulating the insect susceptibility to Cry1 toxins and also to other groups of toxins, such as Cry2 and Cry3*.* Examples of membrane proteins that are known to serve as surrogate receptors for a wide variety of Cry proteins are cadherin, APN and ALP. All have been reported to be the binding proteins for quite different Cry toxins, such as Cry1, Cry3, and Cry4 and Cry11, which are active against Lepidoptera, Coleoptera and Diptera, respectively [10,43-48]. It seems that different Cry proteins have found the appropriate insect species, or even insect order, to interact with a same type of surrogate receptor.

## Conclusions

BSA, based on high-throughput transcriptome sequencing, can be applied for genetic mapping in invertebrates. In this study, BSA has contributed to the identification of a novel type of mutation in the ABCC2 transporter conferring high levels of insect resistance to *B. thuringiensis.* Additional results have expanded the role of the ABCC2 transporter in *B. thuringiensis* resistance beyond the Cry1A family of proteins to include Cry1Ca and revealed the involvement of different members of the ABCC family in the mode of action of *B. thuringiensis* and in the fitness-cost associated with the resistance to *B. thuringiensis.*

## Methods

### Source of Cry toxins

*B. thuringiensis* Cry toxins, Cry1Ac and Cry1Ca, were obtained from recombinant *Escherichia coli* XL-1 strains (kindly supplied by Dr. Ruud de Maagd, Plant Research International, Wageningen, The Netherlands). Protein production, solubilization and trypsin activation was performed as described earlier [49]. Proteins used for bioassays were in the form of protoxin dissolved in carbonate buffer (0.1 M Na_2_CO_3_, 0.01 M ethylenediaminetetraacetic acid (EDTA), 0.17 M NaCl, pH 10.5). For binding assays, Cry1Ca was further purified by anion-exchange chromatography followed by gel filtration on a Superdex-75 column containing 1 mM dithiothreitol (DTT) according to published procedures [50,51], to remove the adsorbed toxin fragments that could interfere in the radiolabeling of the toxin. Protein concentration was measured using the Coomassie protein assay reagent (Bio-Rad, Hercules, CA, USA) with bovine serum albumin (Sigma-Aldrich, Tres Cantos, Spain) as the standard.

### Source of insects and genetic crosses

For genetic and binding analyses, two *S. exigua* laboratory colonies were used. The resistant colony (Xen-R) was highly resistant to the commercial bioinsecticide Xentari™ (based on *B. thuringiensis* subsp. aizawai, Valent Biosciences, Libertyville, IL, USA). This colony has been previously described by Hernández-Martínez *et al.,*[25] and the resistance level was maintained with a constant selection protocol in which neonate larvae were reared for five days on an artificial diet containing Xentari™ [52], at 10 mg per gram of artificial diet. The susceptible control colony (FRA) originated from France and has been maintained for at least 10 years without *B. thuringiensis* exposure. For the functional analysis of *ABCC* genes, a third *S. exigua* colony (KOR) was established from larvae collected from a field population infesting welsh onion in Andong, Korea. Larvae from all colonies were reared on an artificial diet at 25°C and adults were fed with 10% sucrose. Insects were reared on an artificial diet at 25 ± 3°C with 70 ± 5% relative humidity and a photoperiod of 16/8 hours (light/dark).

For resistance mapping, single-pair crosses were performed between females from the Xen-R colony and males from the FRA colony. From the F1 offspring from each family, one male was backcrossed with five Xen-R females. Neonate larvae from the resulting progeny (BC families) were reared for five days on an artificial diet containing Xentari™ (10 mg/g) and then transferred to a normal diet until they reached the fifth instar. Survivors from each BC family were pooled (Sel-BC sample) and used for NGS and BSA. Sel-BC insects must be homozygous for the resistance allele or alleles and it should contain, on average, a 75% genome background of the parental resistant colony.

### RNA extraction and NGS sequencing

For BSA, three samples were processed: Xen-R, FRA, and Sel-BC. For each sample, about 30 early fifth instar larvae midguts were dissected and used for total RNA isolation using Trizol (Invitrogen, Carlsbad, CA, USA) following the manufacturer’s instructions. The quality and quantity of total RNA was determined spectroscopically at 230, 260, and 280 nm. About 20 μg of total RNA from each sample was sent to Macrogen Inc (Seoul, Korea) for mRNA isolation and high-throughput sequencing using the Illumina Hi-Seq2000 paired-end technology.

### Sequence analysis

Hi-Seq2000 reads were analyzed using seq_scrumbs [53] to trim bad quality regions. These reads were assembled with Trinity [54] along with the 454-sequencing reads of the previous transcriptome [28]. To keep only the most informative unigenes, a selection of the assembled unigenes was carried out. For a unigene to be finally selected, it should have to be evaluated as positive for any one of the following criteria: having a CDS (Coding DNA sequence), having a Blastx match against *Bombyx* or *Drosophila* proteins, being expressed with a level higher than 0.25 RPKM (reads per kilo base per million) or being longer than 500 bp.

A SNVs (single nucleotide variants: SNPs and Indels) search was done only on the longest unigene from each defined unigene cluster. The Illumina and 454-sequencing reads were mapped against these reduced unigene sets with bowtie2 [55] and bwa for sequences from the 454-sequencing [56]. SNVs were identified with Varscan2 software [57]. Orthologues for each unigene were identified with a bi-directional Blast search comparison against *B. mori* and *Drosophila melanogaster* protein databases.

### Analysis of the genomic region

The genomic sequence for the ABCC2 region, which includes the mutation in the Xen-R colony, was obtained. Primers flanking the deleted region were designed based on the mRNA sequence from the FRA colony and used for PCR amplification of the genomic region using total DNA from Xen-R and FRA adults. PCR products for both colonies were cloned in the pGEM-T-easy vector (Invitrogen) and several clones were sequenced and assembled to generate the consensus sequence. At least three independent clones were used for each of the sequenced samples.

### Sequence comparison and phylogenetic analysis

Sequences from the ABCC receptors from other insect species used for phylogenetic analyses were retrieved after Blast-search against different databases: NCBI, Silkdb and Manduca Base (Agripest). For the sake of clarity, only representative genes from selected lepidopteran species and from the coleopteran *T. castaneum* were included in the analysis. Amino acid sequences were aligned using ClustalX with default settings [58] and visualized in GeneDoc [59]. Phylogenetic analyses were performed using the neighbor-joining method with 100 bootstraps, using the ClustalX [58] and MEGA5 programs [60]. Phylogenetic trees were visualized with the MEGA5 program. Nucleotide binding domains in the ABCC receptors were identified by comparison against the CDD (conserved domain database) at NCBI [61]. Transmembrane regions in the protein were predicted using TOPCONS online software [62].

### Semiquantitative and quantitative RT-PCR

Total RNA was extracted from whole bodies of different developmental stages or from different tissues from fifth instar larvae with Trizol (Invitrogen) according to the manufacturer’s instructions. Aliquots (1 μg) were incubated at 70°C for three minutes and then used for synthesizing cDNA using an RT-mix kit (Intron, Seoul, Korea). For semiquantitative RT-PCR, the synthesized single-stranded cDNA was used as a template for PCR amplification with gene-specific primers for *SeABCC2* (5′-GAG CCT AAC ATG GCT GAC TAT C-3′ and 5′-CTG CCT ACA TTT GCG TCT ACT-3′) and *SeABCC3* (5′-TCT ATA CGA CCG CTC TCT TAC C-3′ and 5′-CAC CCT GCA CAC CTT CTT AT-3′) with 35 cycles under the following conditions: 20 seconds at 94°C for denaturation, 50 seconds at 55°C for annealing, and one minute at 72°C for extension. A housekeeping gene, *β-actin*, was used as an internal control in each sample for an equivalent of template with actin primers (5′-TGG CAC CAC ACC TTCTAC-3′ and 5′-CAT GAT CTG GGT CAT CTT CT-3′) with 35 cycles under the following conditions: 20 seconds at 94°C for denaturation, 20 seconds at 50°C for annealing, and one minute at 72°C for extension.

The mRNA expression level of *SeABCC2* and *SeABCC3* genes was further confirmed by quantitative RT-PCR (qRT-PCR). qRT-PCR was performed with an Applied Biosystems 7500 real-time PCR system (Applied Biosystems, Foster City, CA, USA) using SYBR® Green Realtime PCR master mix (Toyobo, Osaka, Japan) according to the manufacturer’s instructions with the gene-specific primers described above. Template cDNA from 48 hour dsRNA-treated third instar larvae (see below) was used for the qRT-PCR reaction. After a hot start at 94°C for 10 minutes, qRT-PCR was performed with 40 cycles of one minute at 94°C, 30 seconds at 55°C and 40 seconds at 72°C. The housekeeping gene *β-actin* was used as a control with the primers described above. Each condition was independently replicated three times. Quantitative analysis of *SeABCC2 and SeABCC3* expression was done using the comparative C_T_ (∆∆C_T_) method [63].

#### Preparation and delivery of RNA interference (RNAi) of SeABCC2 and SeABCC3

Template cDNA was amplified with the following primers containing T7 RNA polymerase promoter sequence for *SeABCC2* (5′-TAA TAC GAC TCA CTA TAG GGA GAG GAG CCT AAC ATG GCT GAC TAT C-3′ and 5′-TAA TAC GAC TCA CTA TAG GGA GAG CTG CCT ACA TTT GCG TCT ACT-3′) and *SeABCC3* (5′-TAA TAC GAC TCA CTA TAG GGA GAG TCT ATA CGA CCG CTC TCT TAC C-3′ and 5′-TAA TAC GAC TCA CTA TAG GGA GAG CAC CCT GCA CAC CTT CTT AT-3′). Sequence identity between *SeABCC2* and *SeABCC3* at the region used for dsRNA was less than 65%. The PCR products were used to prepare dsRNA using the MEGA Script RNAi kit according to the manufacturer’s instructions (Ambion, Austin, TX, USA). The synthesized RNAs were annealed at 37°C for four hours and then left at 70°C for five minutes. Before dsRNA feeding, the solution was prepared by mixing a transfection reagent, Metafectene PRO (Biontex, Plannegg, Germany), with dsRNA in 1:1 (v/v) ratio and then incubated for 20 minutes at 25°C.

For the dsRNA feeding assays, cabbage (1 × 1 cm) was used as a carrier by applying 4 μL of the dsRNA (150 ng total) solution on a piece of cabbage leaf. Second instar larvae were starved for six hours before being allowed to feed on cabbage treated with dsRNA for four hours at 25°C. Then, fresh artificial diet was supplied to the larvae to continue development. To determine the effect of the transfection reagent on *S. exigua* larvae, a control bioassay was performed using only 4 μL of Metafectene PRO solution on cabbage leaf. Metafectene PRO does not affect the development and mortality of control larvae (data not shown). Knockdown of *SeABCC2* and *SeABCC3* gene expression was evaluated by RT-PCR at selected periods up to 96 hours post feeding. A viral gene, CpBV-ORF302, was used as a negative control for RNAi [64].

### Bioassays

*S. exigua* third instar larvae were used in bioassays with Cry1Ac and Cry1Ca proteins. For the concentration response bioassays, the concentrations tested for Cry1Ac were 0.625, 1.25, 2.5, 5 and 10 μg/cm^2^, while for Cry1Ca, the concentrations tested were 0.062, 0.125, 0.25, 0.5 and 1 μg/cm^2^. Cry1 toxins were serially diluted with sterile deionized water and then overlaid on the piece of cabbage leaf and air-dried for 20 minutes. Each bioassay was conducted with 10 larvae per replicate and three replicates per concentration. Mortality was scored at five days post-treatment. LC_50_ values were calculated by Probit analysis using the EPA Probit Analysis program, version 1.5 (US Environmental Protection Agency, Cincinnati, OH, USA).

For dsRNA bioassays, *S. exigua* second instar larvae were fed dsRNA specific to *SeABCC2* and *SeABCC3* as described above for 24 hours. Then, larvae were exposed to Cry1Ac or Cry1Ca protoxins for five days using the same method as before. Control larvae were treated with 4 μL of control dsRNA (dsCON). Each treatment was replicated three times with ten larvae per replicate. Means and variances of treatments were analyzed in one-way analysis of variance (ANOVA) by PROC GLM of SAS program (SAS Institute, 1989). The means were compared by the LSD test at Type I error = 0.05.

### Binding assays

Last-instar larvae from the susceptible (FRA) and the resistant (Xen-R) colonies were dissected and the bolus-free midguts were preserved at -80°C until required. BBMVs were prepared by the differential magnesium precipitation method [65], frozen in liquid nitrogen, and stored at -80°C. The total protein concentration of the BBMV preparations was determined by the Bradford method using bovine serum albumin as standard.

Chromatography-purified Cry1Ca was labeled using the chloramine-T method [66]. Cry1Ca (25 μg) was mixed with 0.5 mCi of ^125^NaI (PerkinElmer, Boston, MA, USA), chloramine T was added to be at 6 mM final concentration. After 45 seconds of incubation at room temperature, the reaction was stopped by adding potassium metabisulfite followed by non-radioactive NaI. The specific activity of the ^125^I-Cry1Ca was 0.73 μCi/μg.

All binding assays were performed at room temperature in a final volume of 0.1 ml in binding buffer (phosphate buffer saline: pH 7.4 phosphate-buffered saline (PBS), supplemented with 0.1% bovine serum albumin). For the determination of the optimal concentration of BBMV to be used for the binding assays, 1 nM of ^125^I-Cry1Ca was incubated for one hour with increasing concentrations of BBMV (from either FRA or Xen-R insects). The non-specific binding was determined using an excess of unlabeled toxin.

Competition binding assays were performed by incubating 1 nM of ^125^I-Cry1Ca with 32 μg of BBMV and increasing concentrations of unlabeled toxin for one hour. The unbound radio-labeled toxin was washed out and the amount of residual bound ^125^I-Cry1Ca was determined using a 2480 WIZARD^2^ (Perkin Elmer, Waltham, MA, USA) gamma counter. The dissociation constant (*K*_*d*_) and concentration of binding sites (*R*_*t*_) were calculated using the LIGAND program [67].

To determine the contribution of reversible and irreversible binding to the observed specific binding, three reaction mixtures were prepared with 1 nM of ^125^I-Cry1Ca and 30 μg of BBMV, and all the samples were incubated for two hours. One sample was used to determine the total binding. In a second sample, used to determine the non-specific binding, an excess of unlabeled toxin was added at the start of the incubation. To the third sample, which was used to measure the irreversible binding, an excess of unlabeled toxin was added after one hour of incubation and the reaction was allowed to proceed until two hours. The specific binding was calculated by subtracting the radioactivity in the pellet of the second sample from that in sample one and the irreversible binding was calculated by subtracting the radioactivity in the pellet of the second sample from that in the third sample. Therefore, the reversible binding was calculated by subtracting the irreversible binding from the amount of specific binding. Experiments were performed in triplicate and similar results were obtained from two independent batches of BBMV.

### Developmental analysis of dsRNA treatment

RNAi-treated second instar larvae were analyzed for the possible effects on pupation and adult emergence. Metafectene PRO transfection reagent (4 μL) without dsRNA was used to feed control larvae. Each treatment was replicated three times with ten larvae per replicate. Pupation success, weight of newly pupated individuals, pupation time at 25°C and adult emergence were analyzed.

#### Expression levels of arylphorin and repat5 genes by RNAi of SeABCC2 and SeABCC3 genes

cDNAs prepared from individuals at 48 hours after ds*SeABCC2*, ds*SeABCC3* or dsRNA^*ORF302*^ (dsCON) feeding were used for the analysis of *arylphorin* and *repat5* gene expression by qRT-PCR. After a hot start at 94°C for 10 minuts, qRT-PCR amplification was performed as before with gene-specific primers for *arylphorin* and *repat5*[25].

## Competing interests

The authors declare that they have no competing interests.

## Authors’ contributions

JB, JC and SH designed the study. YP, RGM, GNC, MC, PZ and SH carried out the experiments. JB, JC, YK, JF and SH analyzed the data. JF and SH wrote the paper. All authors read and approved the final manuscript.

## Supplementary Material

Additional file 1 ***S. exigua *** **unigenes with orthologs in *D. melanogaster *** **and *B. mori.***
Click here for file

Additional file 2 **SNVs (single nucleotide variants: SNPs and Indels) from *S. exigua* identified in the BSA studies.** Click here for file

Additional file 3 **Unigenes from *S. exigua* mapping to the *B. mori *** **genome and their BSA index.** Click here for file

Additional file 4: Figure S1 Alignment of the predicted amino acid sequences of ABBCC2 and ABCC3 transporters from *S. exigua*. Blue and orange regions are predicted transmembrane domains for ABCC2 and ABCC3, respectively. The red dotted line localizes the deletion in ABCC2 in resistant insects. Click here for file

Additional file 5: Figure S2 Overexpression of *arylphorin* and *repat5* genes induced by *SeABCC2* or *SeABCC3* suppression in third instar *S. exigua*. The expression of *SeABCC2* or *SeABCC3* was suppressed in third instar larva by feeding double-stranded RNA (dsSeABCC2 or dsSeABCC3, 150 ng per larva). A viral gene (ORF302)-specific dsRNA (dsCON) was used for a dsRNA control. Expression of β-actin confirms the integrity of cDNA preparation. Click here for file

## References

[B1] Kumar S Chandra A Pandey KC *Bacillus thuringiensis* (Bt) transgenic crop: an environment friendly insect-pest management strategy J Environ Biol 2008 12 641 653 19295059

[B2] Sanahuja G Banakar R Twyman RM Capell T Christou P *Bacillus thuringiensis*: a century of research, development and commercial applications Plant Biotechnol J 2011 12 283 300 21375687 10.1111/j.1467-7652.2011.00595.x

[B3] Ferré J Van Rie J MacIntosh SC Romeis J, Shelton AM, Kennedy GG Insecticidal genetically modified crops and insect resistance management (IRM) Integration of Insect-resistant Genetically Modified Crops within IPM Programs 2008 Dordrecht: Springer Science and Business Media 41 85

[B4] Tabashnik BE Brevault T Carriere Y Insect resistance to Bt crops: lessons from the first billion acres Nat Biotechnol 2013 12 510 521 23752438 10.1038/nbt.2597

[B5] Sumerford DV Head GP Shelton A Greenplate J Moar W Field-evolved resistance: assessing the problem and ways to move forward J Econ Entomol 2013 12 1525 1534 24020262 10.1603/ec13103

[B6] Vachon V Laprade R Schwartz JL Current models of the mode of action of *Bacillus thuringiensis* insecticidal crystal proteins: a critical review J Invertebr Pathol 2012 12 1 12 22617276 10.1016/j.jip.2012.05.001

[B7] Pardo-López L Soberón M Bravo A *Bacillus thuringiensis* insecticidal three-domain Cry toxins: mode of action, insect resistance and consequences for crop protection FEMS Microbiol Rev 2013 12 3 22 22540421 10.1111/j.1574-6976.2012.00341.x

[B8] Rodrigo-Simón A Caccia S Ferré J *Bacillus thuringiensis* Cry1Ac toxin-binding and pore-forming activity in brush border membrane vesicles prepared from anterior and posterior midgut regions of lepidopteran larvae Appl Environ Microbiol 2008 12 1710 1716 18223107 10.1128/AEM.02827-07 PMC2268304

[B9] Ferré J Van Rie J Biochemistry and genetics of insect resistance to *Bacillus thuringiensis* Annu Rev Entomol 2002 12 501 533 11729083 10.1146/annurev.ento.47.091201.145234

[B10] Pigott CR Ellar DJ Role of receptors in *Bacillus thuringiensis* crystal toxin activity Microbiol Mol Biol Rev 2007 12 255 281 17554045 10.1128/MMBR.00034-06 PMC1899880

[B11] Gill M Ellar D Transgenic *Drosophila* reveals a functional in vivo receptor for the *Bacillus thuringiensis* toxin Cry1Ac1 Insect Mol Biol 2002 12 619 625 12421420 10.1046/j.1365-2583.2002.00373.x

[B12] Rajagopal R Sivakumar S Agrawal N Malhotra P Bhatnagar RK Silencing of midgut aminopeptidase N of *Spodoptera litura* by double-stranded RNA establishes its role as *Bacillus thuringiensis* toxin receptor J Biol Chem 2002 12 46849 46851 12377776 10.1074/jbc.C200523200

[B13] Gahan LJ Gould F Heckel DG Identification of a gene associated with Bt resistance in *Heliothis virescens* Science 2001 12 857 860 11486086 10.1126/science.1060949

[B14] Xu X Yu L Wu Y Disruption of a cadherin gene associated with resistance to Cry1Ac {delta}-endotoxin of *Bacillus thuringiensis* in *Helicoverpa armigera* Appl Environ Microbiol 2005 12 948 954 15691952 10.1128/AEM.71.2.948-954.2005 PMC546791

[B15] Yang Y Chen H Wu Y Yang Y Wu S Mutated cadherin alleles from a field population of *Helicoverpa armigera* confer resistance to *Bacillus thuringiensis* toxin Cry1Ac Appl Environ Microbiol 2007 12 6939 6944 17827322 10.1128/AEM.01703-07 PMC2074965

[B16] Morin S Biggs RW Sisterson MS Shriver L Kirk CE Higginson D Holley D Gahan LJ Heckel DG Carriere Y Dennehy TJ Brown JK Tabashnik BE Three cadherin alleles associated with resistance to *Bacillus thuringiensis* in pink bollworm Proc Natl Acad Sci U S A 2003 12 5004 5009 12695565 10.1073/pnas.0831036100 PMC154288

[B17] Herrero S Gechev T Bakker PL Moar WJ de Maagd RA *Bacillus thuringiensis* Cry1Ca-resistant *Spodoptera exigua* lacks expression of one of four Aminopeptidase N genes BMC Genomics 2005 12 96 15978131 10.1186/1471-2164-6-96 PMC1184072

[B18] Yang Y Zhu YC Ottea J Husseneder C Leonard BR Abel C Huang F Molecular characterization and RNA interference of three midgut aminopeptidase N isozymes from *Bacillus thuringiensis*-susceptible and -resistant strains of sugarcane borer, *Diatraea saccharalis* Insect Biochem Mol Biol 2010 12 592 603 20685334 10.1016/j.ibmb.2010.05.006

[B19] Tiewsiri K Wang P Differential alteration of two aminopeptidases N associated with resistance to *Bacillus thuringiensis* toxin Cry1Ac in cabbage looper Proc Natl Acad Sci U S A 2011 12 14037 14042 21844358 10.1073/pnas.1102555108 PMC3161562

[B20] Gahan LJ Pauchet Y Vogel H Heckel DG An ABC transporter mutation is correlated with insect resistance to *Bacillus thuringiensis* Cry1Ac toxin PLoS Genet 2010 12 e1001248 21187898 10.1371/journal.pgen.1001248 PMC3002984

[B21] Baxter SW Badenes-Pérez FR Morrison A Vogel H Crickmore N Kain W Wang P Heckel DG Jiggins CD Parallel evolution of *Bacillus thuringiensis* toxin resistance in lepidoptera Genetics 2011 12 675 679 21840855 10.1534/genetics.111.130971 PMC3189815

[B22] Atsumi S Miyamoto K Yamamoto K Narukawa J Kawai S Sezutsu H Kobayashi I Uchino K Tamura T Mita K Kadono-Okuda K Wada S Kanda K Goldsmith MR Noda H Single amino acid mutation in an ATP-binding cassette transporter gene causes resistance to Bt toxin Cry1Ab in the silkworm, *Bombyx mori* Proc Natl Acad Sci U S A 2012 12 E1591 E1598 22635270 10.1073/pnas.1120698109 PMC3382473

[B23] Tanaka S Miyamoto K Noda H Jurat-Fuentes JL Yoshizawa Y Endo H Sato R The ATP-binding cassette transporter subfamily C member 2 in *Bombyx mori* larvae is a functional receptor for Cry toxins from *Bacillus thuringiensis* FEBS J 2013 12 1782 1794 23432933 10.1111/febs.12200

[B24] Heckel DG Learning the ABCs of Bt: ABC transporters and insect resistance to *Bacillus thuringiensis* provide clues to a crucial step in toxin mode of action Pestic Biochem Physiol 2012 12 103 110

[B25] Hernández-Martínez P Navarro-Cerrillo G Caccia S de Maagd RA Moar WJ Ferré J Escriche B Herrero S Constitutive activation of the midgut response to *Bacillus thuringiensis* in Bt resistant *Spodoptera exigua* PLoS One 2010 12 9 10.1371/journal.pone.0012795 PMC2941469 20862260

[B26] Michelmore RW Paran I Kesseli RV Identification of markers linked to disease-resistance genes by bulked segregant analysis: a rapid method to detect markers in specific genomic regions by using segregating populations Proc Natl Acad Sci U S A 1991 12 9828 9832 1682921 10.1073/pnas.88.21.9828 PMC52814

[B27] Magwene PM Willis JH Kelly JK The statistics of bulk segregant analysis using next generation sequencing PLoS Comput Biol 2011 12 e1002255 22072954 10.1371/journal.pcbi.1002255 PMC3207950

[B28] Pascual L Jakubowska AK Blanca JM Cañizares J Ferré J Gloeckner G Vogel H Herrero S The transcriptome of *Spodoptera exigua* larvae exposed to different types of microbes Insect Biochem Mol Biol 2012 12 557 570 22564783 10.1016/j.ibmb.2012.04.003

[B29] Liang Y Patel SS Dean DH Irreversible binding kinetics of *Bacillus thuringiensis* CryIA delta-endotoxins to gypsy moth brush border membrane vesicles is directly correlated to toxicity J Biol Chem 1995 12 24719 24724 7559587 10.1074/jbc.270.42.24719

[B30] Van Leeuwen T Demaeght P Osborne EJ Dermauw W Gohlke S Nauen R Grbi-ç M Tirry L Merzendorfer H Clark RM Population bulk segregant mapping uncovers resistance mutations and the mode of action of a chitin synthesis inhibitor in arthropods Proc Natl Acad Sci 2012 12 4407 4412 22393009 10.1073/pnas.1200068109 PMC3311382

[B31] Tabashnik BE Liu YB Malvar T Heckel DG Masson L Ballester V Ménsua JL Ferré J Global variation in the genetic and biochemical basis of diamondback moth resistance to *Bacillus thuringiensis* Proc Natl Acad Sci U S A 1997 12 12780 12785 9371752 10.1073/pnas.94.24.12780 PMC24215

[B32] Wang P Zhao JZ Rodrigo-Simón A Kain W Janmaat AF Shelton AM Ferré J Myers J Mechanism of resistance to *Bacillus thuringiensis* toxin Cry1Ac in a greenhouse population of the cabbage looper, *Trichoplusia ni* Appl Environ Microbiol 2007 12 1199 1207 17189446 10.1128/AEM.01834-06 PMC1828666

[B33] Gassmann AJ Carriere Y Tabashnik BE Fitness costs of insect resistance to *Bacillus thuringiensis* Annu Rev Entomol 2009 12 147 163 19067630 10.1146/annurev.ento.54.110807.090518

[B34] Navarro-Cerrillo G Ferré J de Maagd RA Herrero S Functional interactions between members of the REPAT family of insect pathogen-induced proteins Insect Mol Biol 2012 12 335 342 22404489 10.1111/j.1365-2583.2012.01139.x

[B35] Navarro-Cerrillo G Hernández-Martínez P Vogel H Ferré J Herrero S A new gene superfamily of pathogen-response (repat) genes in Lepidoptera: classification and expression analysis Comp Biochem Physiol B Biochem Mol Biol 2013 12 10 17 23036664 10.1016/j.cbpb.2012.09.004

[B36] Rodríguez-Cabrera L Trujillo-Bacallao D Borrás-Hidalgo O Wright DJ Ayra-Pardo C Molecular characterization of *Spodoptera frugiperda*-*Bacillus thuringiensis* Cry1Ca toxin interaction Toxicon 2008 12 681 692 18222513 10.1016/j.toxicon.2007.12.002

[B37] Liu JB Tabashnik BE Pusztai-Carey M Field-evolved resistance to *Bacillus thuringiensis* toxin CryIC in diamondback moth (Lepidoptera: Plutellidae) J Econ Entomol 1996 12 798 804

[B38] Hernández-Martinez P Ferré J Escriche B Susceptibility of *Spodoptera exigua* to 9 toxins from *Bacillus thuringiensis* J Invertebr Pathol 2008 12 245 250 18082763 10.1016/j.jip.2007.11.001

[B39] Hernández-Rodríguez CS Hernández-Martínez P Van RJ Escriche B Ferré J Specific binding of radiolabeled Cry1Fa insecticidal protein from *Bacillus thuringiensis* to midgut sites in lepidopteran species Appl Environ Microbiol 2012 12 4048 4050 22447600 10.1128/AEM.07591-11 PMC3346400

[B40] Granero F Ballester V Ferré J *Bacillus thuringiensis* crystal proteins CRY1Ab and CRY1Fa share a high affinity binding site in *Plutella xylostella* (L.) Biochem Biophys Res Commun 1996 12 779 783 8713122 10.1006/bbrc.1996.1099

[B41] Xie X Cheng T Wang G Duan J Niu W Xia Q Genome-wide analysis of the ATP-binding cassette (ABC) transporter gene family in the silkworm, *Bombyx mori* Mol Biol Rep 2012 12 7281 7291 22311044 10.1007/s11033-012-1558-3

[B42] Liu S Zhou S Tian L Guo E Luan Y Zhang J Li S Genome-wide identification and characterization of ATP-binding cassette transporters in the silkworm Bombyx mori. BMC Genomics 2011 12 491 10.1186/1471-2164-12-491 PMC3224256 21981826

[B43] Contreras E Schoppmeier M Real MD Rausell C Sodium solute symporter and cadherin proteins act as *Bacillus thuringiensis* Cry3Ba toxin functional receptors in *Tribolium castaneum* J Biol Chem 2013 12 18013 18021 23645668 10.1074/jbc.M113.474445 PMC3689946

[B44] Zúñiga-Navarrete F Gómez I Pena G Bravo A Soberon M A *Tenebrio molitor* GPI-anchored alkaline phosphatase is involved in binding of *Bacillus thuringiensis* Cry3Aa to brush border membrane vesicles Peptides 2013 12 81 86 22743140 10.1016/j.peptides.2012.05.019

[B45] Jiménez AI Reyes EZ Cancino-Rodezno A Bedoya-Pérez LP Caballero-Flores GG Muriel-Millan LF Likitvivatanavong S Gill SS Bravo A Soberón M *Aedes aegypti* alkaline phosphatase ALP1 is a functional receptor of *Bacillus thuringiensis* Cry4Ba and Cry11Aa toxins Insect Biochem Mol Biol 2012 12 683 689 22728570 10.1016/j.ibmb.2012.06.001 PMC3416946

[B46] Hua G Zhang R Abdullah MA Adang MJ *Anopheles gambiae* cadherin AgCad1 binds the Cry4Ba toxin of *Bacillus thuringiensis* israelensis and a fragment of AgCad1 synergizes toxicity Biochemistry 2008 12 5101 5110 18407662 10.1021/bi7023578 PMC2662694

[B47] Saengwiman S Aroonkesorn A Dedvisitsakul P Sakdee S Leetachewa S Angsuthanasombat C Pootanakit K In vivo identification of *Bacillus thuringiensis* Cry4Ba toxin receptors by RNA interference knockdown of glycosylphosphatidylinositol-linked aminopeptidase N transcripts in *Aedes aegypti* larvae Biochem Biophys Res Commun 2011 12 708 713 21439264 10.1016/j.bbrc.2011.03.085

[B48] Likitvivatanavong S Chen J Bravo A Soberon M Gill SS Cadherin, alkaline phosphatase, and aminopeptidase N as receptors of Cry11Ba toxin from *Bacillus thuringiensis* subsp. jegathesan in *Aedes aegypti* Appl Environ Microbiol 2011 12 24 31 21037295 10.1128/AEM.01852-10 PMC3019721

[B49] Herrero S González-Cabrera J Ferré J Bakker PL de Maagd RA Mutations in the *Bacillus thuringiensis* Cry1Ca toxin demonstrate the role of domains II and III in specificity towards *Spodoptera exigua* larvae Biochem J 2004 12 507 513 15320864 10.1042/BJ20041094 PMC1134136

[B50] Zhao JZ Collins HL Tang JD Cao J Earle ED Roush RT Herrero S Escriche B Ferré J Shelton AM Development and characterization of diamondback moth resistance to transgenic broccoli expressing high levels of Cry1C Appl Environ Microbiol 2000 12 3784 3789 10966391 10.1128/aem.66.9.3784-3789.2000 PMC92221

[B51] Van Rie J Jansens S Höfte H Degheele D Van Mellaert H Receptors on the brush border membrane of the insect midgut as determinants of the specificity of *Bacillus thuringiensis* delta-endotoxins Appl Environ Microbiol 1990 12 1378 1385 2339890 10.1128/aem.56.5.1378-1385.1990 PMC184414

[B52] Xentari Technical bulletin. Valent Biosciences http://microbials.valentbiosciences.com/docs/ag-microbials-resources-library/xentari-protoxin-blend-technical-bulletin-ag-5398

[B53] Public Repository for Seq Crumbs files https://github.com/JoseBlanca/seq_crumbs]

[B54] Haas BJ Papanicolaou A Yassour M Grabherr M Blood PD Bowden J Couger MB Eccles D Li B Lieber M Macmanes MD Ott M Orvis J Pochet N Strozzi F Weeks N Westerman R William T Dewey CN Henschel R Leduc RD Friedman N Regev A De novo transcript sequence reconstruction from RNA-seq using the Trinity platform for reference generation and analysis Nat Protoc 2013 12 1494 1512 23845962 10.1038/nprot.2013.084 PMC3875132

[B55] Langmead B Salzberg SL Fast gapped-read alignment with Bowtie 2 Nat Methods 2012 12 357 359 22388286 10.1038/nmeth.1923 PMC3322381

[B56] Li H Durbin R Fast and accurate short read alignment with Burrows-Wheeler transform Bioinformatics 2009 12 1754 1760 19451168 10.1093/bioinformatics/btp324 PMC2705234

[B57] Koboldt DC Zhang Q Larson DE Shen D McLellan MD Lin L Miller CA Mardis ER Ding L Wilson RK VarScan 2: somatic mutation and copy number alteration discovery in cancer by exome sequencing Genome Res 2012 12 568 576 22300766 10.1101/gr.129684.111 PMC3290792

[B58] Thompson JD Gibson TJ Plewniak F Jeanmougin F Higgins DG The CLUSTAL_X windows interface: flexible strategies for multiple sequence alignment aided by quality analysis tools Nucleic Acids Res 1997 12 4876 4882 9396791 10.1093/nar/25.24.4876 PMC147148

[B59] Nicholas KB Nicholas HBJ Deerfield DW GeneDoc: analysis and visualization of genetic variation EMBNEW NEWS 1997 12 14

[B60] Tamura K Peterson D Peterson N Stecher G Nei M Kumar S MEGA5: molecular evolutionary genetics analysis using maximum likelihood, evolutionary distance, and maximum parsimony methods Mol Biol Evol 2011 12 2731 2739 21546353 10.1093/molbev/msr121 PMC3203626

[B61] Marchler-Bauer A Zheng C Chitsaz F Derbyshire MK Geer LY Geer RC Gonzales NR Gwadz M Hurwitz DI Lanczycki CJ Lu F Lu S Marchler GH Song JS Thanki N Yamashita RA Zhang D Bryant SH CDD: conserved domains and protein three-dimensional structure Nucleic Acids Res 2013 12 D348 D352 23197659 10.1093/nar/gks1243 PMC3531192

[B62] Bernsel A Viklund H Hennerdal A Elofsson A TOPCONS: consensus prediction of membrane protein topology Nucleic Acids Res 2009 12 W465 W468 19429891 10.1093/nar/gkp363 PMC2703981

[B63] Livak KJ Schmittgen TD Analysis of relative gene expression data using real-time quantitative PCR and the 2(-Delta Delta C(T)) method Methods 2001 12 402 408 11846609 10.1006/meth.2001.1262

[B64] Park B Kim Y Transient transcription of a putative RNase containing BEN domain encoded in *Cotesia plutellae* bracovirus induces an immunosuppression of the diamondback moth, *Plutella xylostella* J Invertebr Pathol 2010 12 156 163 20600089 10.1016/j.jip.2010.06.003

[B65] Wolfersberger MG Luthy P Maurer A Parenti P Sacchi VF Giordana B Hanozet GM Preparation and partial characteritzation of amino acid transporting brush border membrane vesicles from the larval midgut of the cabbage butterfly (*Pieris brassicae)* Comp Biochem Physiol A Physiol 1987 12 301 308

[B66] Van Rie J Jansens S Höfte H Degheele D Van Mellaert H Specificity of *Bacillus thuringiensis* delta-endotoxins. Importance of specific receptors on the brush border membrane of the mid-gut of target insects Eur J Biochem 1989 12 239 247 2557209 10.1111/j.1432-1033.1989.tb15201.x

[B67] Munson PJ Rodbard D LIGAND: a versatile computerized approach for characterization of ligand-binding systems Anal Biochem 1980 12 220 239 6254391 10.1016/0003-2697(80)90515-1

