# Supplementary figures and images for: ABCC transporters mediate insect resistance to multiple Bt toxins revealed by bulk segregant analysis
Source: BMC Biol. 2014 Jun 9;12:46. doi: 10.1186/1741-7007-12-46 (PMC4071345; doi:10.1186/1741-7007-12-46)

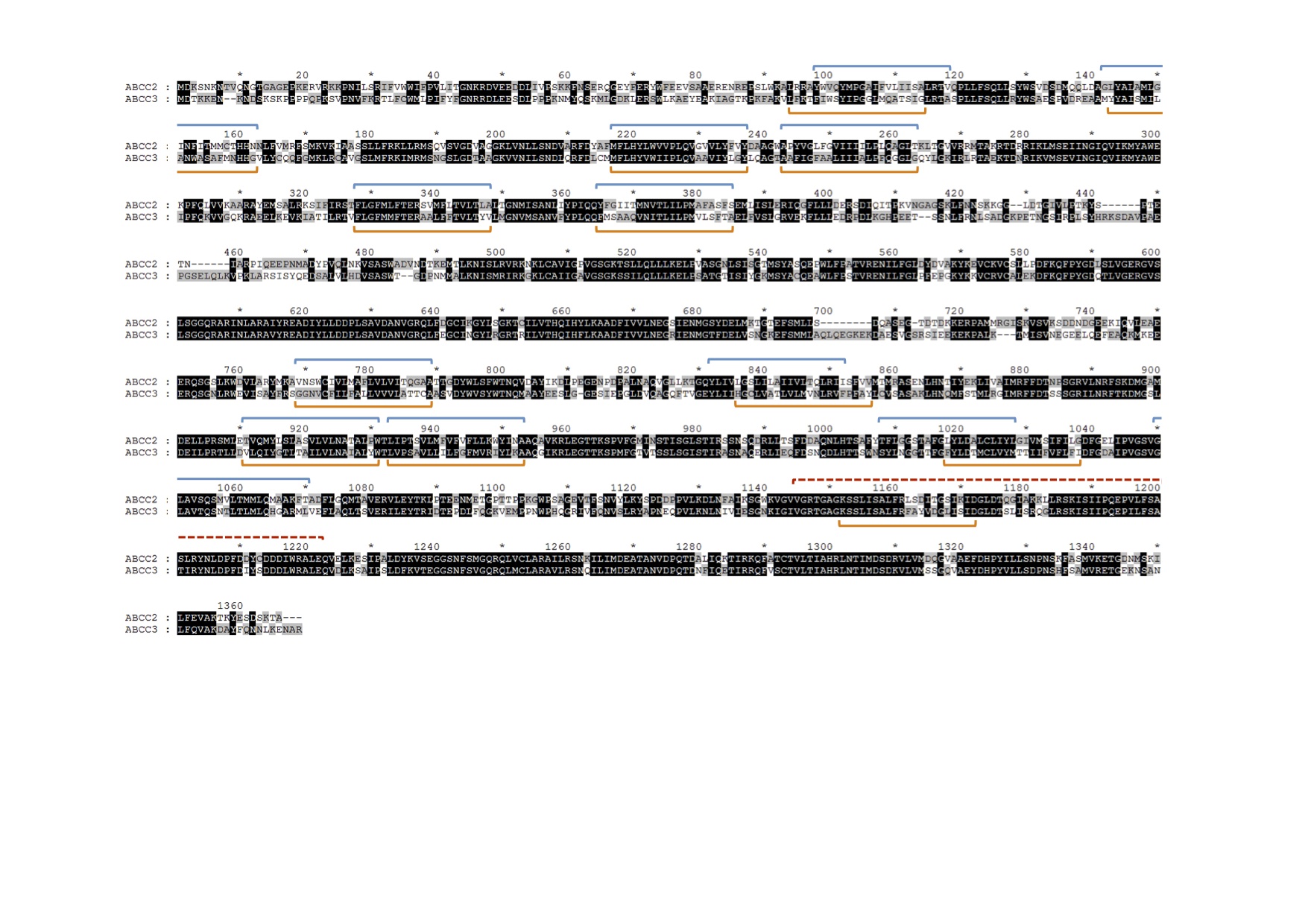

Supplement: Additional file 4: Figure S1 — Alignment of the predicted amino acid sequences of ABBCC2 and ABCC3 transporters from S. exigua. Blue and orange regions are predicted transmembrane domains for ABCC2 and ABCC3, respectively. The red dotted line localizes the deletion in ABCC2 in resistant insects. [file 1741-7007-12-46-S4.jpeg]

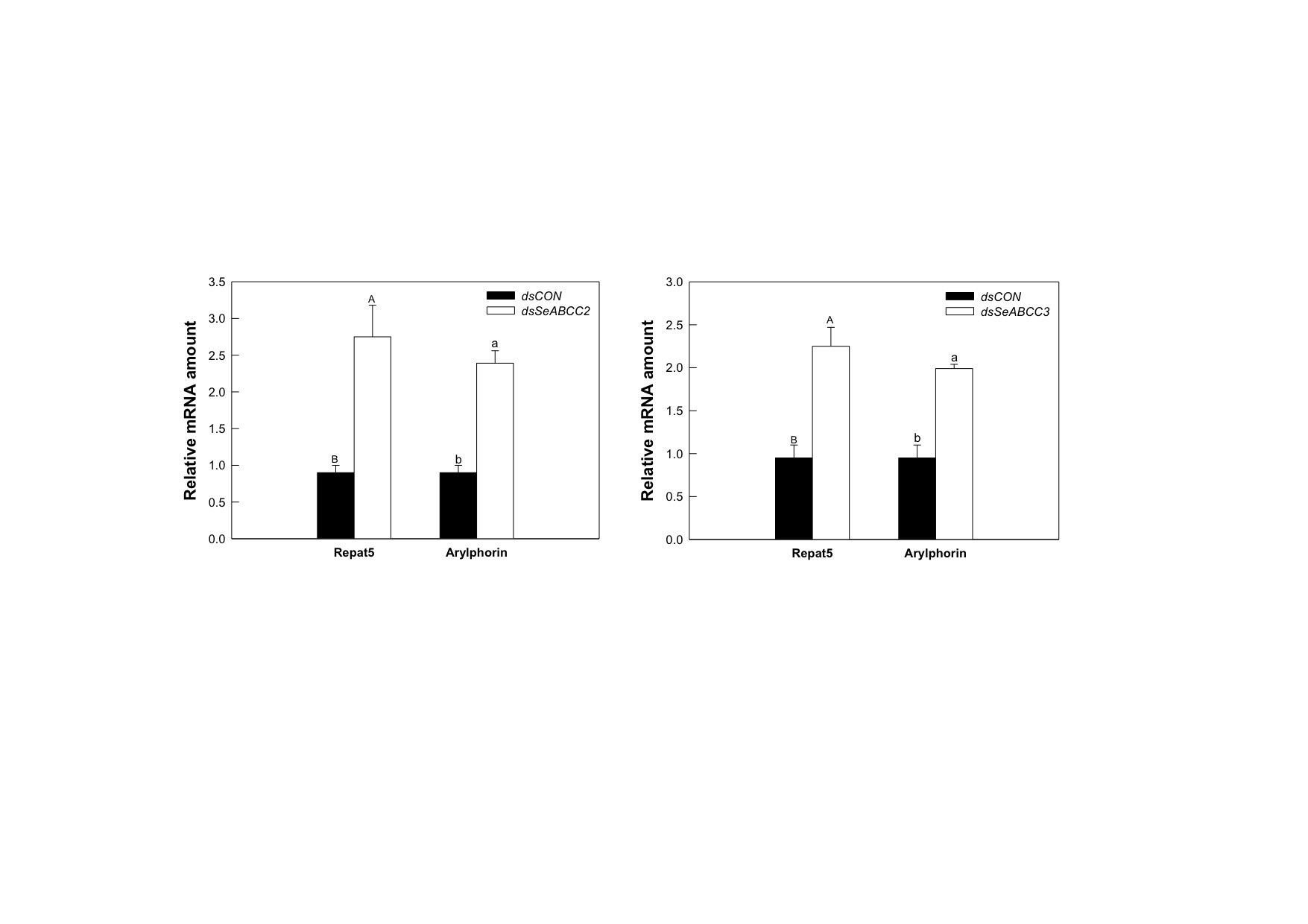

Supplement: Additional file 5: Figure S2 — Overexpression of arylphorin and repat5 genes induced by SeABCC2 or SeABCC3 suppression in third instar S. exigua. The expression of SeABCC2 or SeABCC3 was suppressed in third instar larva by feeding double-stranded RNA (dsSeABCC2 or dsSeABCC3, 150 ng per larva). A viral gene (ORF302)-specific dsRNA (dsCON) was used for a dsRNA control. Expression of β-actin confirms the integrity of cDNA preparation. [file 1741-7007-12-46-S5.jpeg]
